# Obesity, Metabolic Syndrome, and Musculoskeletal Disease: Common Inflammatory Pathways Suggest a Central Role for Loss of Muscle Integrity

**DOI:** 10.3389/fphys.2018.00112

**Published:** 2018-02-23

**Authors:** Kelsey H. Collins, Walter Herzog, Graham Z. MacDonald, Raylene A. Reimer, Jaqueline L. Rios, Ian C. Smith, Ronald F. Zernicke, David A. Hart

**Affiliations:** ^1^Human Performance Laboratory, Faculty of Kinesiology, University of Calgary, Calgary, AB, Canada; ^2^McCaig Institute for Bone and Joint Health, University of Calgary, Calgary, AB, Canada; ^3^Department of Biochemistry and Molecular Biology, University of Calgary, Calgary, AB, Canada; ^4^CAPES Foundation, Brasilia, Brazil; ^5^Departments of Orthopaedic Surgery and Biomedical Engineering, School of Kinesiology, University of Michigan, Ann Arbor, MI, United States; ^6^Department of Surgery, Department of Physiology and Pharmacology, University of Calgary, Calgary, AB, Canada; ^7^Department of Family Practice, The Centre for Hip Health and Mobility, University of British Columbia, Vancouver, BC, Canada; ^8^Alberta Health Services Bone and Joint Health Strategic Clinical Network, Calgary, AB, Canada

**Keywords:** joint diseases, muscle, bone, tendon, NFkB, MAPK

## Abstract

Inflammation can arise in response to a variety of stimuli, including infectious agents, tissue injury, autoimmune diseases, and obesity. Some of these responses are acute and resolve, while others become chronic and exert a sustained impact on the host, systemically, or locally. Obesity is now recognized as a chronic low-grade, systemic inflammatory state that predisposes to other chronic conditions including metabolic syndrome (MetS). Although obesity has received considerable attention regarding its pathophysiological link to chronic cardiovascular conditions and type 2 diabetes, the musculoskeletal (MSK) complications (i.e., muscle, bone, tendon, and joints) that result from obesity-associated metabolic disturbances are less frequently interrogated. As musculoskeletal diseases can lead to the worsening of MetS, this underscores the imminent need to understand the cause and effect relations between the two, and the convergence between inflammatory pathways that contribute to MSK damage. Muscle mass is a key predictor of longevity in older adults, and obesity-induced sarcopenia is a significant risk factor for adverse health outcomes. Muscle is highly plastic, undergoes regular remodeling, and is responsible for the majority of total body glucose utilization, which when impaired leads to insulin resistance. Furthermore, impaired muscle integrity, defined as persistent muscle loss, intramuscular lipid accumulation, or connective tissue deposition, is a hallmark of metabolic dysfunction. In fact, many common inflammatory pathways have been implicated in the pathogenesis of the interrelated tissues of the musculoskeletal system (e.g., tendinopathy, osteoporosis, and osteoarthritis). Despite these similarities, these diseases are rarely evaluated in a comprehensive manner. The aim of this review is to summarize the common pathways that lead to musculoskeletal damage and disease that result from and contribute to MetS. We propose the overarching hypothesis that there is a central role for muscle damage with chronic exposure to an obesity-inducing diet. The inflammatory consequence of diet and muscle dysregulation can result in dysregulated tissue repair and an imbalance toward negative adaptation, resulting in regulatory failure and other musculoskeletal tissue damage. The commonalities support the conclusion that musculoskeletal pathology with MetS should be evaluated in a comprehensive and integrated manner to understand risk for other MSK-related conditions. Implications for conservative management strategies to regulate MetS are discussed, as are future research opportunities.

## Methodology

The studies presented in this review were identified through PubMed Searches and the review of relevant papers in the area of diet-induced obesity, musculoskeletal health, musculoskeletal disease, and inflammation.

## Introduction to metabolic syndrome and obesity

Metabolic syndrome (MetS) is a cluster of conditions—visceral obesity, hypertension, dyslipidemia, and elevated fasting glucose—that increase an individual's risk for diabetes and cardiovascular complications (Alberti and Zimmet, 1998; Manuel et al., 2014). Human metabolism has evolved to efficiently convert chemical energy obtained through the consumption of food into thermal and chemical energy. Our body's metabolic pathways have developed to provide energy to tissues in times of physical threat and survival, or to efficiently conserve energy in times of food deprivation. Today, westernized societies have an abundance of food (food security) and many individuals have little need to perform physical activity. This combination has led to excessive nutrient storage, placing significant stress on our metabolic pathways, and leading to an increase in the prevalence of disease stemming from metabolic dysfunction (Miranda et al., 2005).

**Figure 1:**
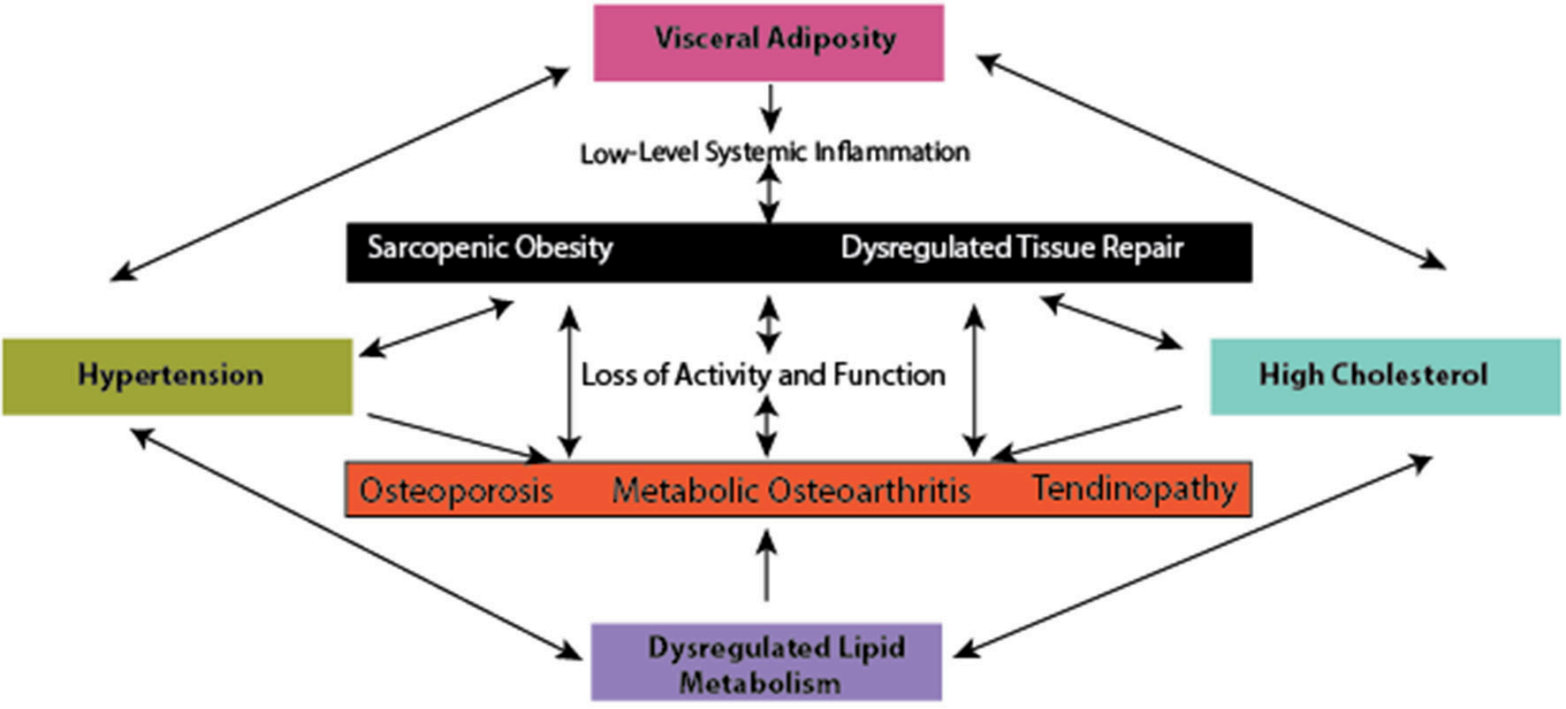
The interface between metabolic complications and musculoskeletal compromise.

Concordant with the rise in MetS prevalence, there is also a global increase in the prevalence of musculoskeletal (MSK) diseases and disorders (Wearing et al., 2006). Recent evidence demonstrates that metabolic complications also increase the risk for the most prominent MSK diseases, such as sarcopenic obesity (muscle loss in obesity), osteoporosis, tendinopathy, and osteoarthritis, conditions which contribute significantly to disability and time lost from work. The resultant damage and pain associated with these conditions likely develops through low-level systemic inflammation (Hoy et al., 2014; Smith et al., 2014; Figure 1) in addition to loading due to obesity (Felson et al., 1988), and reduced ability to withstand loading due to sarcopenia.

MSK disease is of particular concern, for example, as osteoarthritis-related walking disability significantly increases risk for all-cause mortality and cardiovascular events, when controlling for other cofounders (Hawker et al., 2014). This suggests that MSK disability associated with MetS can contribute to the worsening of MetS through sedentary behavior. Although obesity has received considerable attention regarding chronic cardiovascular conditions and diseases, as well as diabetes, the MSK complications that result from obesity associated MetS are less frequently discussed and rarely evaluated comprehensively.

**Figure 2:**
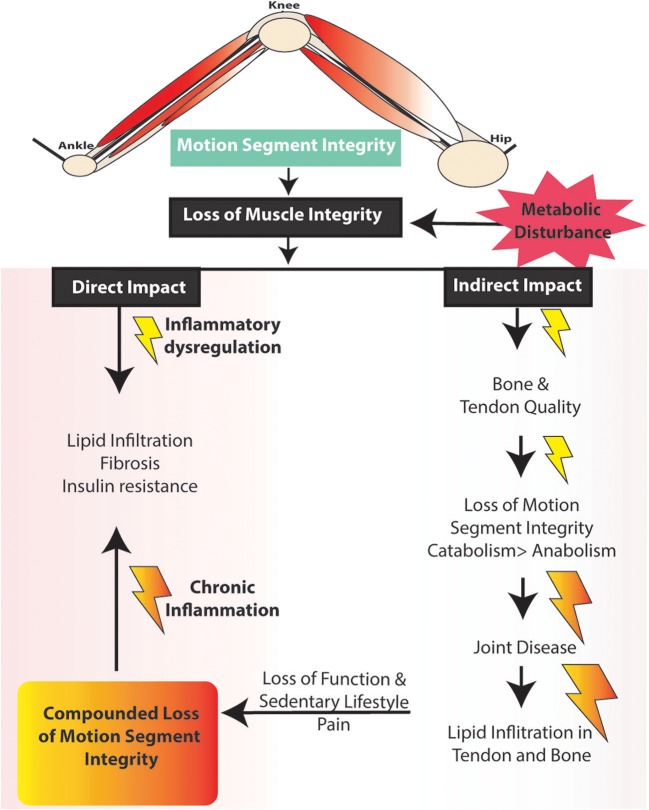
Potential impact of changes in muscle damage on lower limb motion segment integrity.

Muscle mass is a key predictor of longevity in older adults (Srikanthan and Karlamangla, 2014), and since muscle is highly plastic and undergoes regular remodeling, it is a vulnerable tissue in a chronic low-level inflammatory environment, such as that seen with metabolic dysfunction (Tidball, 2005; Fink et al., 2014; D'Souza et al., 2015; Collins et al., 2016a). For example, intramuscular lipid deposits increase with obesity and are also positively correlated with insulin resistance (Akhmedov and Berdeaux, 2013; Addison et al., 2014; Fellner et al., 2014), linking structural alterations with altered capacity for glucose homeostasis. Functionally, impaired muscle integrity, persistent atrophy, and lipid accumulation in muscle are risk factors for tendinopathy (Meyer and Ward, 2016), osteoporosis (Ormsbee et al., 2014), osteoarthritis (Lee et al., 2012), and integrity of a motion segment, such as the leg (Figure 2).

## A role for metabolic disturbance in motion segment tissue damage

**Figure 3:**
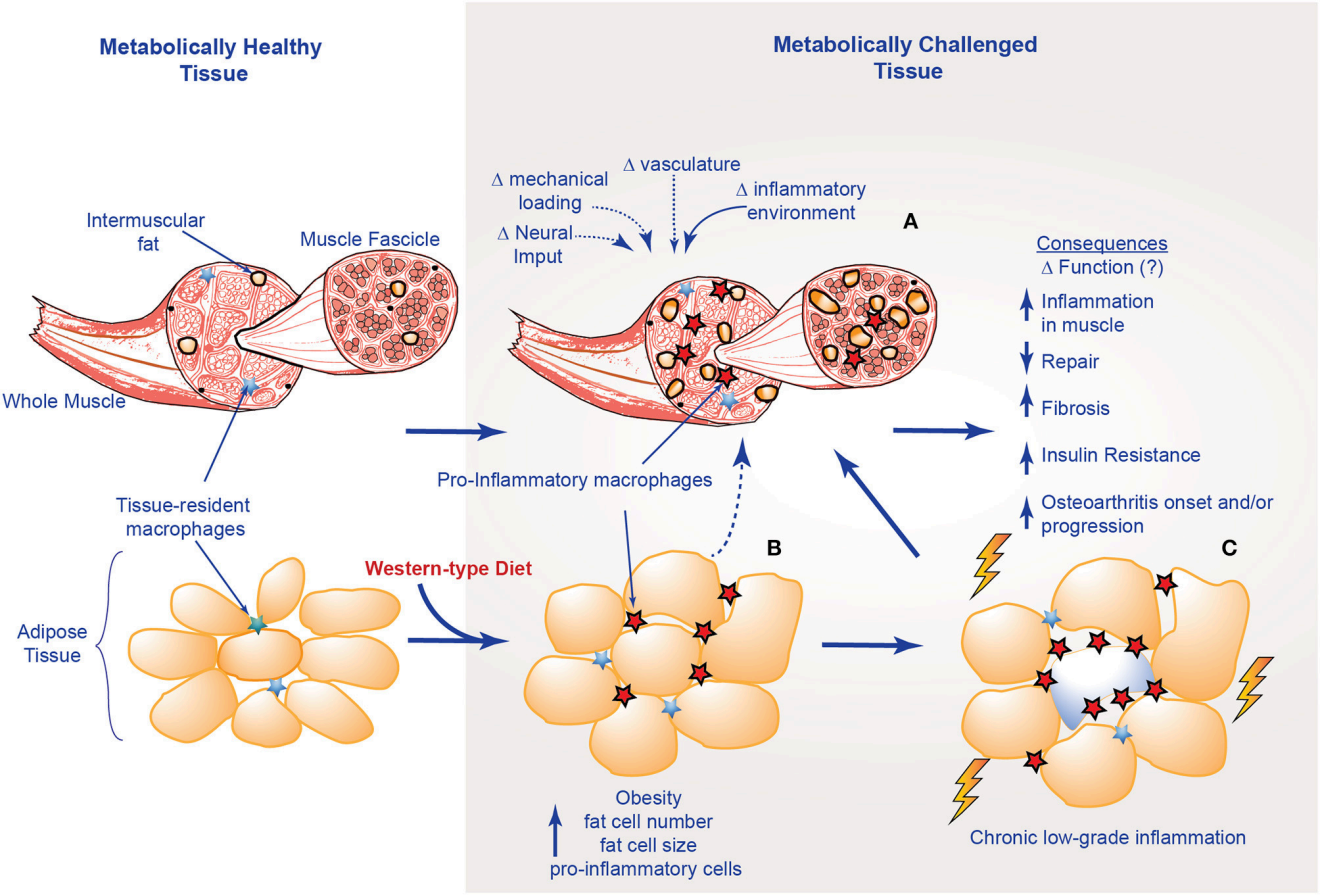
Structural and inflammatory changes in muscle with obesity; **(A)** factors that influence muscle structural integrity with metabolic challenge; **(B)** alterations in adipose tissue; **(C)** musculoskeletal consequences of chronic-low grade inflammation.

A hypothesis has emerged proposing that metabolically mediated damage to MSK tissues may be one additional component of the MetS, as adipose-based inflammation links obesity, MetS, and MSK tissue damage (Hart and Scott, 2012; Zhuo et al., 2012). Increased visceral adiposity is linked to induction of increased levels of catabolic mediators (Fontana et al., 2007) and ultimately tissue damage (Figures 1 – 3). Additionally, the presence of hypertension may be linked to tissue damage through vasoconstriction and ultimately depriving tissues of appropriate nutrient exchange (McMaster et al., 2015). Moreover, high cholesterol is speculated to link dysregulated lipid metabolism and endothelial dysfunction and has been directly associated with tissue damage in tendons (Tilley et al., 2015), decreased bone mineral density (Makovey et al., 2009), and osteoarthritis damage (Farnaghi et al., 2017). In parallel, investigations into the impact of low-level systemic inflammation from metabolic disturbance on the onset of sarcopenic obesity tendinopathy, osteoporosis, and osteoarthritis have been conducted. Many of these authors report associations between low-level systemic inflammation from diet-induced obesity (DIO) with MSK disease outcomes (Table 1), however these diseases are seldom evaluated comprehensively to evaluate a common inflammatory pathway to disease induction and progression.

**Table 1:** Examples of MSK damage resulting from DIO.

**Species**	**Strain**	**Experimental diet**	**MSK changes observed due to DIO**	**Authors**	**Year**
Mouse	C57b6	60% Fat	Quadriceps muscle macrophages—short term	Fink	2014
			Quadriceps muscle macrophages	Patsouris	2008
			Metabolic knee OA	Griffin	2012
			Decreased tendon failure stress load	Grewal	2014
			Decrease in bone quality and quantity	Ionova-Martin	2010
Mouse	C57b6	40% Fat	Increased macrophages in soleus muscle	Nguyen	2007
Mouse	C57b6	45% Fat	Decreased cancellous bone mass	Cao	2009
Mouse	C57b6	10% Corn oil	Osteoporosis outcomes	Halade	2010
Mouse	C57b6	45% Fat, 40% Sucrose	Decreased BMD, uCT and osteoporosis outcomes	Bhatta	2016
			Adverse effect on bone morphology and mechanics	Lorincz	2010
Rat	Sprague dawley	40–45% Fat, 45–40% Sucrose	Compromised vastus lateralis muscle integrity in 3-days	Collins	2016
			Fibrosis and lipid deposition in VL	Collins	2016
			Metabolic knee OA	Collins	2015a, 2015b, 2016, 2017a
Rabbit	New Zealand white	50% Fat	Increased metabolic knee OA	Brunner	2012
		1% Cholesterol, 3% Peanut oil	Increased knee joint tissue damage	Prieto-Potin	2013
Pig	Ossabaw	20% Fructose, 46% Fat, 20% Fructose, 2% Cholesterol	Muscle damage	Clark	2011
Monkey	Rhesus	42% Fat, 27% sucrose	Muscle myosin heavy chain transition from oxidative to glycolytic isoforms	Hyatt	2016

As such, we suggest a hypothesis linking altered muscle integrity to direct and indirect consequences on the motion segment (Figures 2, 3). An obesogenic diet, resulting in over nutrition and development of a low-level systemic inflammation, acutely challenges associated tissues of the motion segment and can result in positive adaptation (i.e., through dynamic compensation that helps the tissue accommodate metabolic challenge), as well as negative adaptation (i.e., vulnerability to deleterious changes in tissue integrity). With these initial challenges, such tissue adaptive responses are balanced and help preserve the associated tissues and motion segment integrity. However, with chronic exposure to obesogenic diet and its inflammatory consequences, tissues demonstrate dysregulated repair, an imbalance toward negative adaptation resulting in regulatory failure, tissue damage (i.e., ectopic lipid storage and tissue fibrosis), and failure of motion segment integrity progressing to loss of function and ultimately, disease.

## Aim and scope of review

The aim of this review is to summarize the links between induction of local and systemic inflammation, DIO, and a central role for muscle integrity in the inflammation-based pathogenesis of these MSK diseases. Early loss of muscle integrity can have both direct and indirect “ripple” effects downstream on tendons and bones, as well as the functioning of multi-tissue complex joints (e.g., the joint as an organ, Figure 2) (Frank et al., 2004; Loeser et al., 2012). As muscle plays a critical role in the mechanical and biological homeostasis of bones (through the muscle tendon unit indirectly and directly through the muscle/bone interface), tendons (directly through the muscle tendon unit), and joints (through loading and stabilization), we suggest that with the influence of dysregulated muscle loading, inflammation, and altered muscle integrity, failure of motion segment integrity is induced and exacerbated.

This review is focused on outcomes from studies using pre-clinical DIO models, as the gradual and progressive pathway toward MetS afforded by DIO provides critical insight into the short- and long-term pathophysiology, in addition to the phenotype of MSK diseases (Buettner et al., 2007; Nilsson et al., 2012). Evaluating diet-induced alterations allows for linking results across systems from food, through the gut and the associated microbiome, to early and late tissue-based changes, as well as the inclusion of potential epigenetic outcomes regarding temporal relations for the onset and progression of MSK disease.

## Obesity and impact on muscle integrity

### Sarcopenic obesity

Sarcopenic obesity, or low muscle mass and quality with increased fat mass, is not only associated with poor physical function (Zamboni et al., 2008), but also results in additional weight gain and an 8 to 11-fold increase in the risk for three or more additional physical disabilities (Baumgartner, 2000). Sarcopenic obesity was first defined clinically using two criteria: (a) an individual who is −2 standard deviations in muscle mass index (muscle mass/height^2^) compared to healthy, same-sex younger individuals; and (b) an individual who has a body fat percentage greater than the median body fat percentage for each sex (males: muscle mass index <7.26 kg/m^2^ with body fat percentage >27%, females: muscle mass index <5.45 kg/m^2^ body fat percentage >38%) (Baumgartner et al., 1998; Baumgartner, 2000). An alternative criteria for sarcopenic obesity involves falling below a linear regression-based threshold amount of lean mass given an amount of fat mass (Stenholm et al., 2008).

Sarcopenic obesity can predict disability and loss of activity in elderly adults (Baumgartner et al., 2004), and sarcopenic obesity is more closely associated with MetS than either obesity or sarcopenia alone. This suggests important roles for both fat accumulation and muscle loss in the etiology of MetS (Lim et al., 2010). Although the age-dependent declines in muscle structure and strength are well-documented, the mechanism by which MetS results in sarcopenic obesity remains to be clarified (Kob et al., 2014). As sarcopenic obesity results in disability, loss of activity, altered mechanical loading, and altered biological function in the muscle due to lipid deposition and its sequelae (Ormsbee et al., 2014), it is likely that sarcopenic obesity is central to the development of other musculoskeletal pathologies. In fact, data from our laboratory in a rat model revealed changes in the integrity of specific muscles as early as 3-days on a high-fat/high-sucrose (HFS) diet (Collins et al., 2016c), and correspond to long-term changes in systemic inflammation and gut microbiota (Collins et al., 2015a). These data support the notion that muscle may be among the first MSK tissues affected by DIO, and inflammation likely plays a substantial role in this loss of integrity.

### Dysregulated tissue regeneration of muscle—primary tissue damaged by MetS

Muscle fiber damage happens on a daily basis and is generally considered to be a beneficial stimulus, leading to growth and adaptation through muscle regenerative processes (Karalaki et al., 2009). In muscle, monocyte and macrophage recruitment, as well as phagocytosis of necrotic material, occurs within the first 24 hours. Muscle is repaired through a series of tightly-controlled inflammatory processes (Akhmedov and Berdeaux, 2013). Specifically, muscle regeneration is a multistep process involving degeneration, regeneration, and remodeling, ultimately restoring structure and function (Laumonier and Menetrey, 2016).

The three most active cells in the regeneration of skeletal muscle are macrophages, satellite cells, and fibroblasts (Akhmedov and Berdeaux, 2013). The metabolic complications associated with obesity can result in an inappropriate temporal recruitment of these cells, which in turn leads to impaired angiogenesis and myocyte formation, while promoting the deposition of fibrotic and adipose tissue, ultimately leading to a reduction in structural integrity and functional capacity of a muscle (Karalaki et al., 2009).

With DIO, metabolic dysfunction and the presence of chronic low level inflammation can impair the “normal” inflammatory response and the regenerative capacities of skeletal muscles, resulting in a pseudo-injury (Collins et al., 2016c; Figure 3). For example, elevated levels of leptin, a satiety hormone now appreciated to have a role in low-level systemic inflammation, can impair angiogenesis, leading to tissue ischemia (Brown et al., 2015). IL-6 expression at the muscle level is a key mediator of macrophage infiltration and muscle repair (Zhang et al., 2013), while elevated expression of TGF-β promotes an increased fibrotic tissue deposition (Laumonier and Menetrey, 2016).

Efficient muscle regeneration can be attributed to satellite cells being readily available, and the cells' ability to re-establish residual pools to support multiple rounds of regeneration (Karalaki et al., 2009). Satellite cells are limited by the complex physiological environment in which they interact, an environment that can be significantly altered in individuals with obesity (D'Souza et al., 2015). A pathological host environment can limit a satellite cell's ability to be activated, proliferate, and differentiate into a muscle fiber (D'Souza et al., 2015; Meng et al., 2015). This was elegantly shown by Boldrin and colleagues through the transplantation of satellite cells from mdx mice, a genetic mouse model of muscular dystrophy, into a neutral environment (Boldrin et al., 2015). Despite the impairment of mdx satellite cells as a result of being in the pathogenic environment of an mdx mouse, following transplantation, mdx satellite cells were fully capable of being activated, and could proliferate and differentiate into a fully functional muscle fiber (Boldrin et al., 2015). Macrophages may also inhibit satellite cell activity, suggesting another mechanism by which low-level systemic inflammation may inhibit muscle repair (Tidball and Villalta, 2010).

Impairments in satellite cell activity have been reported to promote fibro/adipogenic progenitor cells (FAP) that normally aid in muscle regeneration, to differentiate into fibroblasts and/or adipocytes (Chapman et al., 2016). FAPs have also been demonstrated to be a source of intramuscular lipid deposition with rotator cuff/supraspinatus tendon injury (Liu et al., 2016). FAPs are thought to be vulnerable to reprogramming in the presence of low-level inflammation, which may result in increased lipid and fibrosis deposition, and may limit reversibility of fibrosis (Mann et al., 2011). Thus, chronic obesity and associated MetS, with inflammation and fatty infiltration of muscles, may lead to a compromise in the regeneration of muscle integrity.

In addition to the environmental challenges to the muscle regeneration posed by metabolic disturbance, skeletal muscle from individuals with obesity displays a greater number of glycolytic-fibers vs. oxidative-fibers when compared to a healthy individual (Pattanakuhar et al., 2017). Since oxidative-fibers generally contain a greater number of satellite cells relative to glycolytic fibers (Karalaki et al., 2009), individuals with obesity may also have fewer satellite cells. Based on this information, impairments in satellite cell function as a result of alterations in cell metabolism, a reduction in cell number, suppression of cell activation, depleted cell reserves, and impaired cell proliferation and differentiation may lead to impairments in muscle fiber regeneration (Akhmedov and Berdeaux, 2013).

**Figure 4:**
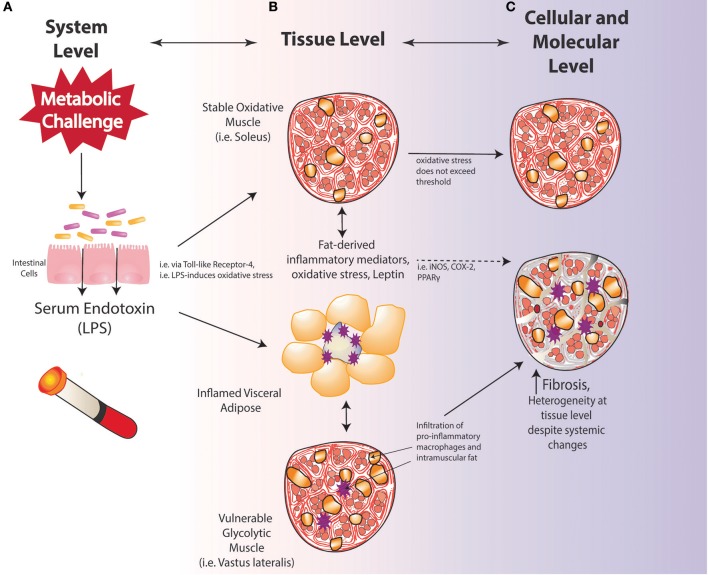
Vulnerability and protection of muscle with diet-induced obesity may be determined by oxidative capacity; **(A)** system level changes; **(B)** tissue-level changes; **(C)** cellular and molecular level alterations.

Work from our laboratory has demonstrated that the oxidative soleus muscle is protected against HFS-induced damage over short-term and long-term exposures in a rat model (Collins et al., 2017b; Figure 4). By 3-days on HFS, dynamic increases in mRNA levels for superoxide dismutase (SOD2) in HFS animals implicate compensatory oxidative stress scavenging in the soleus muscle compared to control animals. By 2-weeks on HFS, increased mRNA levels for oxidative capacity [succinate dehydrogenase (SDH)] were detected compared to chow-fed controls, suggesting one adaptation strategy that the soleus muscle may employ with HFS metabolic challenge. Although the precise mechanism(s) by which the soleus is protected from metabolic disturbance-induced muscle damage remains to be clarified, it appears that increasing the oxidative capacity and the oxidative stress scavenging ability of muscles (i.e., with aerobic exercise) may be a beneficial strategy for mitigating obesity-induced muscle damage and its consequences.

Inflammation related to obesity can also impair myocyte remodeling as a result of a reduction in protein synthesis due to elevated TNF-α levels (Brown et al., 2015). Furthermore, there is evidence for adipocyte-muscle cross talk *in vitro*, whereby adipocyte-derived inflammation can contribute to inflammation and atrophy in muscle cells subjected to a metabolically dysfunctional environment, possibly through IGF-1 (Pellegrinelli et al., 2015). Impaired protein synthesis can also prevent muscle from properly adapting to mechanical stimuli. Brown et al. (2015) demonstrated this inability, showing that following muscle damage, obese muscle displayed no adaptations, while lean mice displayed an increase in muscle wet weight and muscle fiber hypertrophy (Brown et al., 2015). Potential contributing factors to a reduction in protein synthesis may be elevated lipid metabolites resulting from impaired mitochondrial function contributing to elevated TNF-α levels, which are known to have inhibitory effects on IGF-1 (Akhmedov and Berdeaux, 2013). Decreases in IGF-1 result in the inhibition of the muscle growth signaling pathway (IGF-1 → P13K → Akt → mTOR), effectively blunting muscle protein synthesis (Brown et al., 2015). Furthermore, increased myostatin levels can also contribute to impaired growth in obese muscle. Myostatin is not only significantly up-regulated in obese skeletal muscle, but in adipose tissue as well, further inhibiting myogenesis, providing another avenue through which potential muscle-adipose cross talk may occur (Karalaki et al., 2009).

### Functional muscle damage with metabolic derangement

Generally speaking, adults with obesity are reported to have significantly higher absolute strength in lower limb muscles, but lower strength when normalized to body mass (Tomlinson et al., 2016). When the upper limb muscles are evaluated, there are no statistical significant differences between individuals with obesity and normal-weight controls (Tomlinson et al., 2016). Potentially, the characterization of obesity by body mass, which is common in these studies, may be inappropriately representing body composition. We have shown, in a healthy and overweight population cohort, that body mass index (BMI) inappropriately estimates body composition in 30% of the population, with a specific disparity between BMI and body composition measurements in healthy females (Collins et al., 2017c). Also, there is a lack of data describing the effect of obesity on muscle integrity, and a lack of consistent protocols to assess muscle strength (Tomlinson et al., 2016). However, data from our lab (Collins et al., 2016a,c, 2017b) and others, in rodents (Ciapaite et al., 2015) and large mammals (Clark et al., 2011), have demonstrated deleterious alterations in muscle structural integrity with metabolic disturbance. Computational approaches modeling the gastrocnemius muscle have demonstrated that whole-muscle force is dependent on muscle integrity, specifically regarding reductions of muscle force due to intramuscular lipid (Rahemi et al., 2015). A zebrafish model of diet-induced obesity further demonstrated that obesity induces decreases in locomotor performance, isolated muscle isometric stress, work-loop power output, and muscle relaxation rates (Seebacher et al., 2017). Of note, these decrements in performance and function were not reversed with weight loss, generating interesting questions about the potential reversibility of impaired muscle function with obesity.

**Figure 5:**
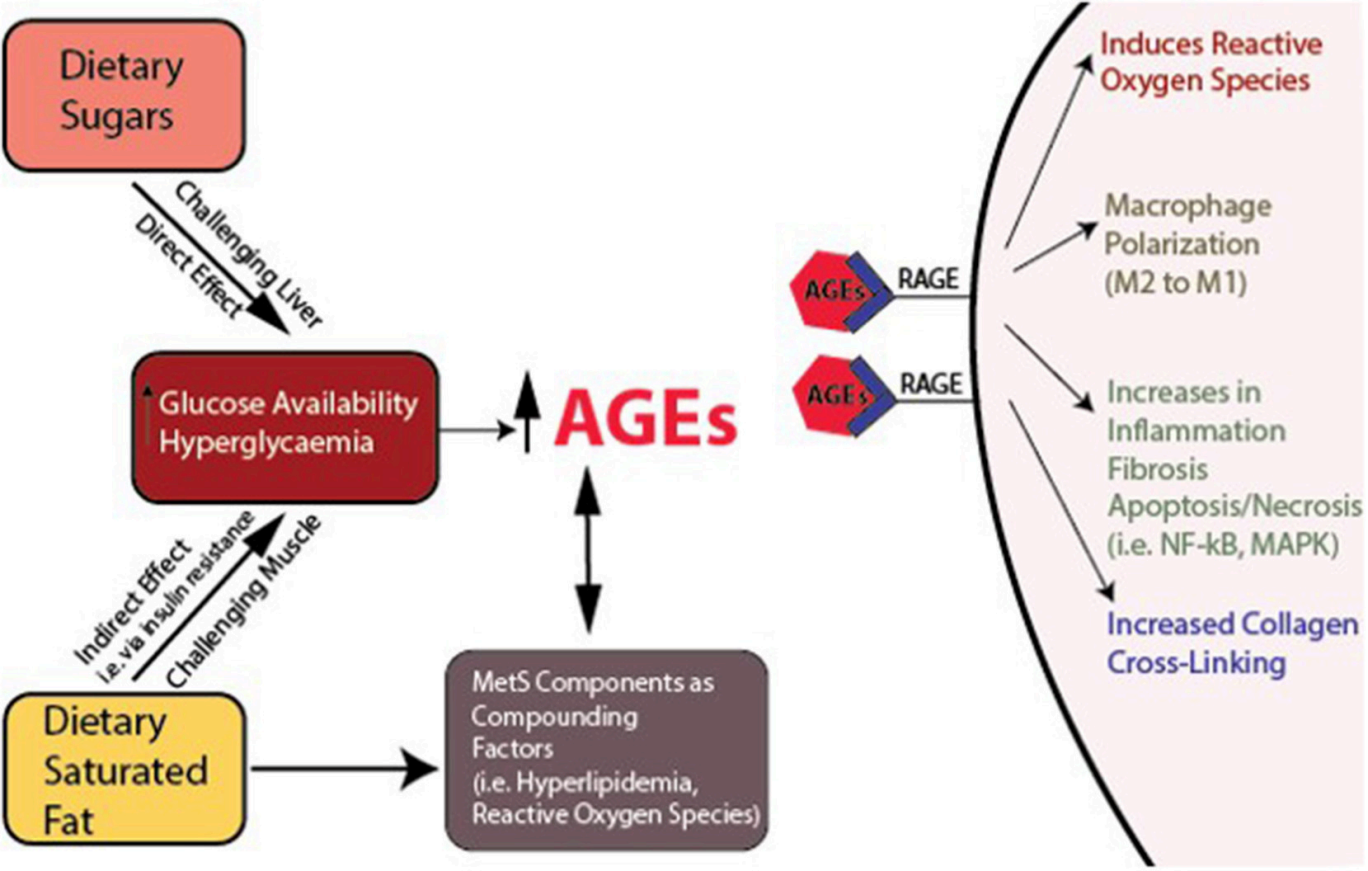
Interacting variables reinforcing metabolic dysfunction and sequelae. Links between dietary sugar, dietary saturated fat, increased hyperglycaemia, advanced glycation end products (AGEs), their receptors (RAGEs), and inflammation, macrophage polarization, and collagen cross-linking.

Additional sources of muscle damage and altered repair are advanced glycation end products (AGEs), which accumulate over time due to increased availability of glucose and hyperglycemia (Figure 5). Dietary AGEs can interfere with muscle healing and impair contractile function in a mouse model of obesity (Egawa et al., 2017). HFS diets can induce hyperglycemia in rodents, further linking diet-induced obesity to AGEs (Sumiyoshi et al., 2006). The receptor for AGEs on macrophages, called RAGE, is associated with a pro-inflammatory state, and RAGE/AGEs are reported to be involved in the onset and progression of metabolic disturbance, insulin resistance, and adipokine expression (Leuner et al., 2012; Hofmann et al., 2014). AGEs have been implicated in macrophage polarization toward M1 pro-inflammatory phenotypes, pro-inflammatory IL-6 secretion in adipose tissues, and initiating inflammatory cascades (Bopp et al., 2008; Frommhold et al., 2011; Nativel et al., 2013; Jin et al., 2015; Son et al., 2016) [i.e., NF-κB, p38 mitogen-activated protein kinase (MAPK)]. Reactive AGEs can also cross-link with collagen fibers, which subsequently can affect the fiber's mechanical and biological properties (Abate et al., 2013). Although AGE collagen cross-linking can be reversed (Asif et al., 2000), it is unclear whether glycation itself can be reversed. Of note, endurance exercise has been shown to attenuate AGEs in cardiac muscle of rats (Wright et al., 2014). With weight loss, reversal of metabolic dysfunction may not be fully achievable, and weight re-gain is common (Fothergill et al., 2016). As mentioned above, to what degree reversibility of skeletal muscle damage may be achieved in this context remains to be determined (Figures 2 – 4), and several factors could contribute to irreversibility (Mann et al., 2011). Likely, clarifying relations between the time of exposure to low-level systemic inflammation and muscle damage should be determined, as impaired muscle integrity has been observed following short-term exposure to a metabolic challenge, likely before “full” metabolic derangement has been achieved (Fink et al., 2014; Collins et al., 2016c). Also, some alterations in satellite cell function can be irreversible (Sacco and Puri, 2015). Generally, DNA methylation, or epigenetic changes, is an actively researched area being explored in the context of muscle (Carrió and Suelves, 2015), and DNA methylation is an important step in muscle cell differentiation (Brunk et al., 1996). However, it is likely that epigenetic changes, which are dynamic and induced by environmental changes, can induce cellular reprogramming, and thus could potentially limit the reversibility of muscle damage, even if the MetS-related inflammation is controlled (Carrió and Suelves, 2015). Investigations to clarify the role of methylation in physiological and pathophysiological muscle changes may provide valuable insight into the reversibility potential of muscle damage.

### Dysregulated homeostasis of associated motion segment tissues (tendon, bone, cartilage, and joint) with obesity: acute and chronic

Muscle, tendon, cartilage, and bone tissues repair and regenerate over different timelines, and these different repair timelines may dictate vulnerability or resistance to damage with metabolic disturbance involving inflammatory processes. Both biological and mechanical stimuli are critical to homeostatic tissue regulatory mechanisms. Across tissues, remodeling rates slow with age. As discussed above, muscle tissue is likely the most vulnerable MSK tissue to perturbation with metabolic challenge. Repair of such tissues in the face of an active inflammatory environment is likely compromised, and resolution of repair is particularly challenging (Hart et al., 2004).

Altered muscle integrity can challenge the motion segment in a dynamic manner, until the associated tissues ultimately begin to fail (Figures 2, 3). Specifically, tendon healing is slower compared to muscle. In tendon, tenocytes migrate to the wound, initiating type III collagen synthesis. After several weeks to months, remodeling of tendinous tissue occurs such that collagen fibers are aligned in the direction of stress, and maturation occurs over the course of a year. Bone mechanotransduction, initiating the maintenance/remodeling processes (Scott et al., 2008), requires a series of events that can require almost 24-h to ultimately result in the synthesis of bone matrix proteins (Robling and Turner, 2009). Cartilage is considered to have limited intrinsic repair properties, although with the facilitation of autologous stem cells, biologics, and other transplants, some repair may be achievable (Chu et al., 2010). The impaired regenerative capacity of tendon, bone and cartilage likely indicate a lack of reversibility with altered integrity and subsequent compromise in function, underscoring a need for strategies aimed at primary prevention (before it occurs), secondary prevention (limiting the amount of damage as it occurs), tertiary prevention (prevention of progression of damage to disease), or reversal of damage.

## Diet-induced obesity and elements of the dysregulated inflammatory response

### Inflammatory mediator alterations in obesity

#### Cytokines and chemokines

Activation of the innate immune system is critical in the pathogenesis of type 2 diabetes and tissue damage. Some of the key signaling pathways involved in this process are nuclear factor –κB (NF-κB), c-Jun N-terminal Kinase (JNK), and the NLRP3 inflammasome, all resulting in transcription of pro-inflammatory cytokines (Lackey and Olefsky, 2015). NF-κB can be activated by elevated levels of pro-inflammatory cytokines such as TNF-α, which is increased in the adipose tissue of obese and diabetic animals. Neutralizing TNF-α has been shown to reduce insulin resistance (Hotamisligil et al., 1993). Additionally, JNK activity increases in tissues that are sensitive to insulin, is activated by ER stress, and directly inhibits insulin signaling (Gual et al., 2005; Lackey and Olefsky, 2015). The inflammasome is a protein complex that matures and secretes inflammatory cytokines, such as IL-1β and IL-18. Similar to NF-κB, the inflammasome can be activated by pro-inflammatory cytokines, lipopolysaccharide (LPS), and some forms of low density lipoproteins (LDLs). LPS, low density lipoproteins, and the AGE-product of LDLs can signal through Toll-like Receptor-4 (TLR-4) (Hodgkinson et al., 2008), activating IL-1β and IL-18 signaling.

TLR-4 recognition of saturated fatty acids is necessary to enable NF-κB signaling and induce expression of pro-inflammatory cytokines (TNF-α, IL-6, and MCP-1) (Jialal et al., 2014; Lackey and Olefsky, 2015). TLRs sense pathogen-associated molecular patterns and damage-associated molecular patterns (PAMPs), and regulate the inflammatory responses to mitigate tissue repair (Lee et al., 2013). TLR-4 gene expression and protein content are increased in muscle from patients with obesity and diabetes, potentially contributing to insulin resistance, as well as compromised muscle integrity observed with metabolic disturbance (Reyna et al., 2008). In the context of OA, TLRs can modulate the catabolic pathways and maintain joint homeostasis (Houard et al., 2013). Of interest, the lubricating molecule proteoglycan-4 (PRG-4) has been shown to modulate the inflammatory response through competitive inhibition of TLR-4 receptors, suggesting that as PRG-4 concentration is reduced with OA severity, inflammatory modulation may also be reduced, contributing to the progression of OA (Iqbal et al., 2016). In tendinopathy, there may not be a clear role for TLRs, as catabolic processes in Achilles tendinopathy seem to occur independently of TLR4-induced gene expression from IL-1β or TNF-α (de Mos et al., 2009). In bone, LPS-induced activation of TLR-4 in neutrophils is reported to upregulate the catabolic RANKL osteoclast cascade, linking TLR based inflammation to increased bone resorption (Chakravarti et al., 2009).

Appropriate levels of pro-inflammatory cytokines are necessary for tissue homeostasis. However, exogenous exposure to pro-inflammatory cytokines, or endogenous high levels of pro-inflammatory cytokines, are associated with damage across all musculoskeletal tissues. In muscle, TNF-α, IL-1β, and IL-6 activate transcription of MuRF-1 and MAFBx/atrogin-1, the key muscle atrophy pathway, through IGF/Akt-1 (Akhmedov and Berdeaux, 2013). For example, IL-6 and TNF-α inhibit bone-forming osteoblast cells, and NF-κb, RANKL, TNF-α, IL-6, M-CSF, and MCP-1 can contribute to osteoclast recruitment, maturation, and inhibit osteoclast apoptosis (Roy et al., 2016). In tendon, pro-inflammatory cytokines (IL-1β, TNF-α, IL-6) can disrupt tissue homeostasis, by inducing extracellular matrix degradation, induce other pro-inflammatory cytokines, which result in necrotic and apoptotic cell changes, and affect collagen and elastin expression (Schulze-Tanzil et al., 2011). Cytokine effects in tendon are modulated by mechanical loading in this complex mechano-biological environment (Killian et al., 2012).

The knee joint is a complex organ system with many resident inflammatory cells, particularly in the synovium and infrapatellar fat pad. These tissues secrete pro-inflammatory cytokines, like IL1-α and IL-1β (Sanchez-Adams et al., 2014). In humans, systemic and synovial fluid inflammatory profiles can differentiate between patients based on OA severity, suggesting that cytokine levels may help define OA pathogenesis (Heard et al., 2013). In addition to the infrapatellar fat pad, there is also a synovial adipose depot, and these two depots differ significantly from each other in lean individuals with OA (Harasymowicz et al., 2017). Moreover, infrapatellar fat pads and synovium adipose depots differed in adipocyte size, fibrosis, and macrophage infiltration in OA patients with obesity compared to lean OA patients (Harasymowicz et al., 2017). High glucose concentrations can contribute to increased chondrocyte responsiveness to cytokines, increased levels of reactive oxygen species, leading to an overproduction of IL-6 and PGE2 (Laiguillon et al., 2015). These cytokines can also contribute to mitochondrial dysfunction within the joint, and, in turn, mitochondrial dysfunction can amplify chondrocyte responsiveness to cytokines (Vaamonde-García et al., 2012).

**Figure 6:**
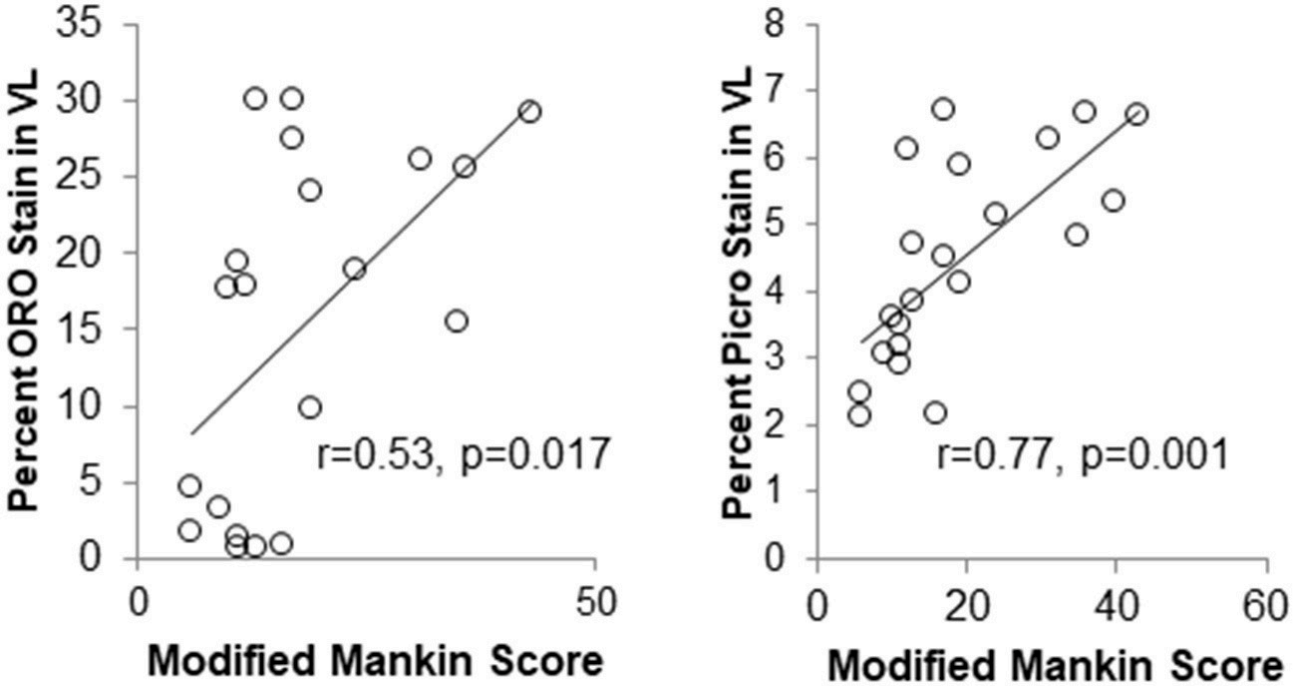
Markers of muscle integrity are associated with metabolic OA severity after 12-weeks of high-fat high-sucrose diet-induced obesity. (ORO, Oil Red O stain for intramuscular fat; Picro, Picrosirius stain for collagen).

Unpublished data from the author's laboratories suggest that intramuscular lipid deposits and fibrotic material in the vastus lateralis muscle of the knee are associated with the presence of metabolic-induced knee joint damage after a 12-week obesity induction period (Figure 6). Based on the inflammatory mediator profile of joints undergoing damage (Collins et al., 2015a,b, 2016b), inflammation is likely playing a significant role in this process and in the relations between muscle damage and joint damage. Ongoing efforts will probe the role of associated inflammation on muscle integrity in the onset and progression of such metabolic joint damage and whether it leads to overt OA.

Given the systemic-to-local hypothesis in metabolic OA (Zhuo et al., 2012; Collins et al., 2016b; Berenbaum et al., 2017), we have evaluated hip, knee, and shoulder joints using the HFS rat model system for 12-weeks (Collins et al., 2017a). A HFS diet, in the absence of trauma, resulted in significant increases in joint damage/OA-like changes in the shoulder and knee joints of rats after a standard 12-week obesity induction period. The hip joint, however, was not significantly affected by DIO, which is consistent with findings from human epidemiological studies. Total joint damage, assessed by adding the individual Modified Mankin Scores across all three joints, was increased in DIO animals compared to chow-fed animals, and was associated with the percentage of body fat. Positive significant predictive relations for total joint score were found between body fat and two serum mediators (IL-1α and VEGF). These data suggest that systemic inflammatory alterations from DIO in this model system may result in a higher incidence of knee, shoulder, and multi-joint OA-like/joint damage with the HFS diet over the long term (Collins et al., 2017a). Due to the preliminary nature of these studies, longitudinal experiments with multiple time points are needed to validate these proposed relations. If these relations are supported in future studies, then not all joints are affected equally by obesity-associated inflammation in MetS. Studying these relations are an ongoing direction of current research.

#### Adipokines

Adipocytes release adipokines such as leptin, adiponectin, visfatin, and resistin as a signaling mechanism in addition to passively storing energy, which can cause and exacerbate chronic low-level systemic inflammation (Gomez et al., 2009). Adipokines have been shown to induce pro-inflammatory mediators in activated CD4+ T cells from osteoarthritis patients, demonstrating that systemic mediators may play a role in osteoarthritis (Scotece et al., 2017). After interaction with such activated CD4+ T cells, chondrocytes demonstrate increased expression of MMP-13 and decreased expression of collagen-2 and aggrecan.

Leptin is a satiety hormone that increases in a near-linear fashion to body fat (Friedman and Halaas, 1998). It inhibits appetite and regulates body weight, energy expenditure, and maintains glucose homeostasis. However, with metabolic disturbance, individuals become leptin resistant. Leptin is a class-1 cytokine secreted from adipose tissue, and high concentrations of leptin are associated with musculoskeletal tissue damage (Zabeau et al., 2003; Collins et al., 2015a,b, 2016b,c). Leptin activates downstream pro-inflammatory pathways (IL-2, IFNγ) and inhibits the anti-inflammatory pathway (IL-4) (Lechler et al., 1998). Aside from being produced by adipose tissue, leptin levels can be increased by TNF-α, IL-1, and LPS, creating a positive-feedback loop with low-level chronic inflammation (Grunfeld et al., 1996).

Leptin signaling is critical to conventional muscle maintenance (Akhmedov and Berdeaux, 2013), and basal levels of satellite cells are reduced in animals with impaired leptin signaling (Peterson et al., 2008). However, hyperphysiological levels of leptin also stimulate the proliferation and activation of macrophages, which may be a mechanism by which leptin concentration influences tissue damage (Santos-Alvarez et al., 1999). Leptin may also positively upregulate myostatin (Rodríguez et al., 2015), a member of the TGF-β superfamily that negatively regulates muscle mass and growth, and a molecule which is up-regulated in muscle from individuals with metabolic disturbance (Hittel et al., 2009). Upregulation of follistatin has been an effective treatment for muscle degenerative diseases by increasing muscle growth due to its ability to inhibit myostatin, a negative regulator of muscle mass. Follistatin also alleviates synovitis and mitigates OA-like changes from inflammatory arthritis in mice (Yamada et al., 2014). As muscle has a role in OA pathogenesis, protecting both muscles and joints with follistatin represents an attractive therapeutic opportunity for MetS-induced musculoskeletal damage.

Leptin also may be involved in mediating the pathogenesis of osteoarthritis in humans (Fowler-Brown et al., 2014) and other animals (Griffin et al., 2009, 2010, 2012; Collins et al., 2015a,b, 2016b). Leptin is found in synovial fluid of humans (Lübbeke et al., 2013) and other animals (Collins et al., 2015a,b, 2016b). Unpublished mRNA data (Table 2); from our laboratory indicate increased mRNA expression levels for leptin in the fat pad and synovium of rats with DIO compared to those on a chow diet, after a 12-week obesity induction period.

**Table 2:** Differential mRNA levels for leptin, IP-10 and IL-1β with 12- and 28-weeks of diet-induced obesity.

**Length of metabolic challenge**	**Infrapatellar fat pad**			**Synovium**		
	**Leptin**	**IP-10**	**IL-1β**	**Leptin**	**IP-10**	**IL-1β**
12-weeks	13.9 ± 3.1^**^	1.6 ± 0.6	0.60 ± 0.10	7.3 ± 2.6^*^^#^	0.4 ± 0.1	1.0 ± 0.2
28-weeks	0.7 ± 0.1	5.3 ± 1.6^**^	2.9 ± 0.6^**^	1.0 ± 0.4	3.3 ± 0.9^*^	0.9 ± 0.1

*Data are shown as fold change ± standard error. DIO n = 12–14; chow n = 5–7*.

* *Indicates p < 0.05 vs. control*;

** *Indicates p < 0.01 vs. control*,

# *Indicates different in SF between DIO and chow*.

Additionally, leptin may be involved in mediating the pathogenesis of osteoarthritis in humans (Fowler-Brown et al., 2014) and other animals (Griffin et al., 2009, 2010, 2012; Collins et al., 2015a,b, 2016b). Leptin is found in synovial fluid of humans (Lübbeke et al., 2013) and other animals (Collins et al., 2015a,b, 2016b). Increased mRNA levels for leptin were accompanied by increased leptin in the serum and synovial fluid (Collins et al., 2016b). After 28-weeks of DIO, the fat pad and synovium demonstrated disparate up-regulation of IL-1β, although IL-1β was detected in the synovial fluid of these animals (Collins et al., 2015a). These findings suggest that the fat pad and synovium contribute to increased pro-inflammatory synovial fluid profiles (Collins et al., 2016b). However, cartilage explants are not substantially damaged when exposed to physiological levels of leptin (Griffin et al., 2010), despite reports of associations between leptin and MMPs (Koskinen et al., 2011), calling into question the direct involvement of leptin in eliciting cartilage damage.

In the context of bone, leptin influences the formtion and resorption of mineralized tissue by increasing the activity of osteoclasts through RANKL (Ducy et al., 2000). However, there are also reports that leptin supports bone growth and bone MSC differentiation into osteoblasts (Thomas et al., 1999), so the precise mechanism for leptin's effects on bone is unclear, but likely is dependent on exposure and dose (Kawai et al., 2009). Low-level systemic inflammation and leptin can negatively influence tendon structural integrity (Abate, 2014; Abate et al., 2016), can result in accelerated heterotopic ossification in tendon tissues (Jiang et al., 2017) and may be associated with increases in tendon ruptures (Ji et al., 2010). Taken together, increases in systemic leptin—or mediators downstream of leptin—appear to have deleterious effects on all major musculoskeletal tissues. These data support the notion that catabolic activity as a result of increased leptin is an important pathway to clarify in the context of global musculoskeletal integrity (Griffin et al., 2009). More details regarding the interface among leptin signaling, inflammation, metabolism, and musculoskeletal disorders are detailed elsewhere (Abella et al., 2017b).

Resistin is an adipokine involved in insulin resistance, inflammation, and energy homeostasis. Serum resistin levels are reported to be derived from visceral adipose tissue (Milan et al., 2002). There is conflicting evidence as to whether resistin is associated with bone mineral density (Mohiti-Ardekani et al., 2014). Within the joint, synovial fluid levels of resistin are associated with inflammatory and catabolic molecules in the joints of human osteoarthritis patients (Koskinen et al., 2014). However, resistin and visfatin, demonstrated positive predictive relations with recovery from upper extremity soft tissue disorders such as tendinopathy, and are thought to be related to anti-inflammatory response mechanisms (Rechardt et al., 2014).

Adiponectin, another adipokine, is derived from visceral fat, and is thought to serve a protective role on cardiovascular health and glucose homeostasis (Milan et al., 2002). As body fat decreases, adiponectin levels generally increase, and adiponectin may modulate adipose tissue regulation via NF-κB (Ajuwon and Spurlock, 2005). Specifically, adiponectin treatment in obese mice increases bacterial clearance and hematopoietic progenitor proliferation in bone marrow (Masamoto et al., 2016). Adiponectin was shown to be significantly correlated with bone mineral density in a group of osteoporotic and healthy patients (Mohiti-Ardekani et al., 2014). Adiponectin has also been proposed as a systemic biomarker of OA. Plasma adiponectin was significantly higher in a population of OA patients and was also higher in women with erosive hand OA compared to patients with non-erosive OA (Filková et al., 2009). In a separate cohort of hand OA patients, the individuals with the highest levels of adiponectin demonstrated a decreased risk for hand OA progression (Yusuf et al., 2011). However, the study populations were not the same in these two reports (i.e., total numbers, males and females vs. females alone, and use of European populations potentially differing genetically) and therefore, additional research needs to be performed to better evaluate this relationship. Additionally, in a study evaluating 76 males in Thailand with knee OA revealed that levels of adiponectin in synovial fluid correlated with disease severity (Honsawek and Chayanupatkul, 2010). In muscle, adiponectin has been shown to increase fatty acid oxidation and glucose uptake, and to attenuate local inflammation (Nigro et al., 2015). Adiponectin has been proposed as a treatment for diabetic tendinopathy (Rothan et al., 2013), suggesting it may be protective and have favorable anti-inflammatory effects across MSK tissues.

Progranulin is a more recently identified adipokine that may also have anti-inflammatory characteristics. mRNA levels for progranulin are increased in cartilage, synovium, and infrapatellar fat pads from OA patients, and mRNA levels are increased in response to pro-inflammatory stimulation (Abella et al., 2016). Specifically, progranulin has been shown to counteract pro-inflammatory molecule expression (i.e., NOS, MMP-13) induced by IL-1β and the LPS-TLR-4 axis (Abella et al., 2016). Attstrin, a progranulin-derived peptide, is a promising therapeutic candidate for osteoarthritis (Abella et al., 2017a) given that progranulin can counteract IL-1 driven inflammation through TNFR1 in human chondrocytes, and intraarticular injection of progranulin-derived attstrin prevented OA-progression in a surgical model of murine OA (Xia et al., 2015).

### Obesity and involvement of cells of the inflammatory system

#### Macrophages

Generally speaking, macrophages are dichotomized into M1 (pro-inflammatory) and M2 (anti-inflammatory) phenotypes based on their activity during tissue repair processes (Novak and Koh, 2013). However, it is understood that this dichotomy represents an oversimplification based on *in vitro* data which may not accurately represent *in vivo* states (Martinez and Gordon, 2014). Macrophages are described to demonstrate a high degree of functional plasticity and their phenotypes can change based on environmental stimuli (Stout and Suttles, 2004). It is likely useful to consider macrophage activation as a spectrum rather than a binary categorization (Mosser and Edwards, 2008), but for the purposes of this review, and concordant with the current musculoskeletal literature, macrophage activity and these relations are generalized using the M1/M2 paradigm.

Macrophages are derived from monocyte precursor cells. Tissues have resident macrophages, which are responsible for general tissue maintenance. These macrophages are described as alternatively activated, or M2 macrophages, and are induced by TGF-β, IL-4, and IL-13 (Gordon and Martinez, 2010). M1 macrophages (classically activated macrophages) are key phagocytes within tissues, and are induced through IFN-γ activation and LPS-induced TLR signaling or from detection of pathogen-associated molecular patterns (Lampiasi et al., 2016). With obesity, stress, loading, or tissue and niche specific control mechanisms, M2-type macrophages may experience a phenotypic shift. This shift may be influenced by exposure to certain cytokines through their general signaling mechanisms or in the presence of other conditions, including IL-6 (Braune et al., 2017) or TNF-α (Wu et al., 2015). AGEs can also play a role in M2 to M1 polarization (Jin et al., 2015) (Figure 5). Typically, an imbalance in the ratio of M1:M2 macrophages is considered maladaptive, creating an imbalance toward tissue degradation and an absence of adequate repair. MAPK is a key pathway in macrophage-mediated inflammatory responses and may play a significant role in diseases mediated by macrophages (Yang et al., 2014).

Within 24 hours following muscle damage, thousands of macrophages have infiltrated the damaged tissue (Tidball, 2005; Grounds, 2011). Pro-inflammatory M1-macrophages first perform cell lysis, removing necrotic muscle tissue debris (Laumonier and Menetrey, 2016). Anti-inflammatory M2-macrophages infiltrate the damaged site once M1-macrophages have removed necrotic debris (~48 hours following injury), helping resolve the inflammatory response while promoting myogenesis (Akhmedov and Berdeaux, 2013). Through the secretion of a number of growth factors, macrophages promote angiogenesis (FGF, TGF-β), synthesis of ECM proteins (TGF-β), and activation of satellite cells, all promoting myogenesis (Grounds, 2011).

It is possible that an increased presence of M1 macrophage cells in a tissue is an early sign of disrupted tissue homeostasis and repair. With diet-induced obesity, M1-type macrophages are present in the quadriceps muscle of mice (Fink et al., 2014), rats (Collins et al., 2016c), and humans (Fink et al., 2014; Khan et al., 2015). In bone, osteoclasts are considered M1-type macrophages. With a high-fat diet, bone loss is accelerated in young mice due to increased osteoclastogenesis (Shu et al., 2015), and as noted, pro-inflammatory cytokines increase osteoclast activity in humans with obesity through activation of the NF-κB/RANKL pathway (Cao, 2011). In the context of the joint as an organ (Frank et al., 2004), the infrapatellar fat pad does not demonstrate inflammation or M1 polarization prior to knee OA in mice (Barboza et al., 2017). Synovial membrane samples from OA patients reveal markers for M1 macrophages (IL-1α, IL-1β, and TNF-α) and decreased levels of the M2 marker IL-1RA (Smith et al., 1997). As such, it is challenging to determine if levels of M1/M2 macrophages are a cause or consequence of disease. In rabbits, intra-articular injections of the pro-inflammatory mediators IL-1 and TNF-α stimulate M1 activation and degrade cartilage, suggesting macrophage polarization may occur in synovial cells (Pettipher et al., 1986; Henderson and Pettipher, 1989). Conditional macrophage depletion in obese mice with a knee injury does not mitigate OA severity (Wu et al., 2017b). Moreover, in mice where macrophages were depleted, there was an increase in neutrophils and CD3+ T cells compared to control animals (Wu et al., 2017b). These data suggest that a potential redundancy may exist between macrophages and other inflammatory cells. Macrophages may modulate inflammatory homeostasis within the joint OA and obesity, and that a basal level of macrophages are likely needed to maintain joint health (Wu et al., 2017b). While this study is not conclusive, it demonstrates that the relations among macrophage polarization and presence in obesity and OA are complex. It is important to note that what constitutes a healthy or maladaptive balance in macrophage phenotype in one tissue or disease may differ from another tissue or disease process. As such, these findings are likely context- and tissue-dependent. More studies are needed to better understand whether synovial macrophage polarization, mitigation/modulation, or ablation is a promising therapeutic target in OA pathogenesis (Sun et al., 2016).

## Specific cell involvement in metabolic overload with obesity

Many cells (e.g., adipocytes, hepatocytes, and myocytes) contribute to the conversion of food into chemical energy and can be placed under significant metabolic stress when exposed to a typical western-style diet. Significant increases in the postprandial flux of metabolic substrates results in drastic nutrient spikes characterized by elevated glucose, free fatty acid (FFA), and triglyceride (TG) levels (Weiss et al., 2013). Chronic nutrient overload places metabolic pathways under significant stress, overwhelming subcutaneous adipose depots, leading to adipocyte mitochondrial dysfunction and impaired insulin sensitivity (Miranda et al., 2005). Normally, insulin inhibits lipolysis and promotes glucose transport. However, during chronic metabolic overload, mitochondrial dysfunction can disrupt adipocyte insulin signaling pathways, leading to impaired glucose transport into adipocytes and the inability to suppress cell lipolysis, further increasing blood glucose and free fatty acid (FFA) levels in circulation (Miranda et al., 2005). As a result of increased circulating blood glucose and FFA levels, hepatocytes and myocytes are placed under great metabolic stress.

### Mitochondrial dysfunction and adipose adaptations with metabolic challenge

With the onset of metabolic challenge, the imbalance of redox states and mitochondrial dysfunction may be instrumental to the development of insulin resistance and MetS (Long et al., 2015). With nutrient overload, mitochondrial dysfunction in adipose tissue will give rise to an increase in inflammatory mediators (adipokines) resulting in tissue remodeling, and potentially cell death (Trayhurn, 2005; Vernochet et al., 2014). Adipokine dysregulation can result in the fibrosis, or inadequate healing response, of adipose tissue (Kwon et al., 2012), as well as liver (Chiang et al., 2011). As adipose depots become inflamed and fibrotic, they become limited in their energy storing and endocrine function, resulting in the deposition of ectopic lipid in other tissues coupled with the presences of insulin resistance (i.e., muscle and liver) (Sun et al., 2013). Altered mitochondrial function, ectopic lipid formation, and incomplete or inadequate healing responses are hallmarks of MSK diseases, and are likely initiated by low-level systemic inflammation.

### Mechanisms of tissue metabolic dysfunction with obesity

**Figure 7:**
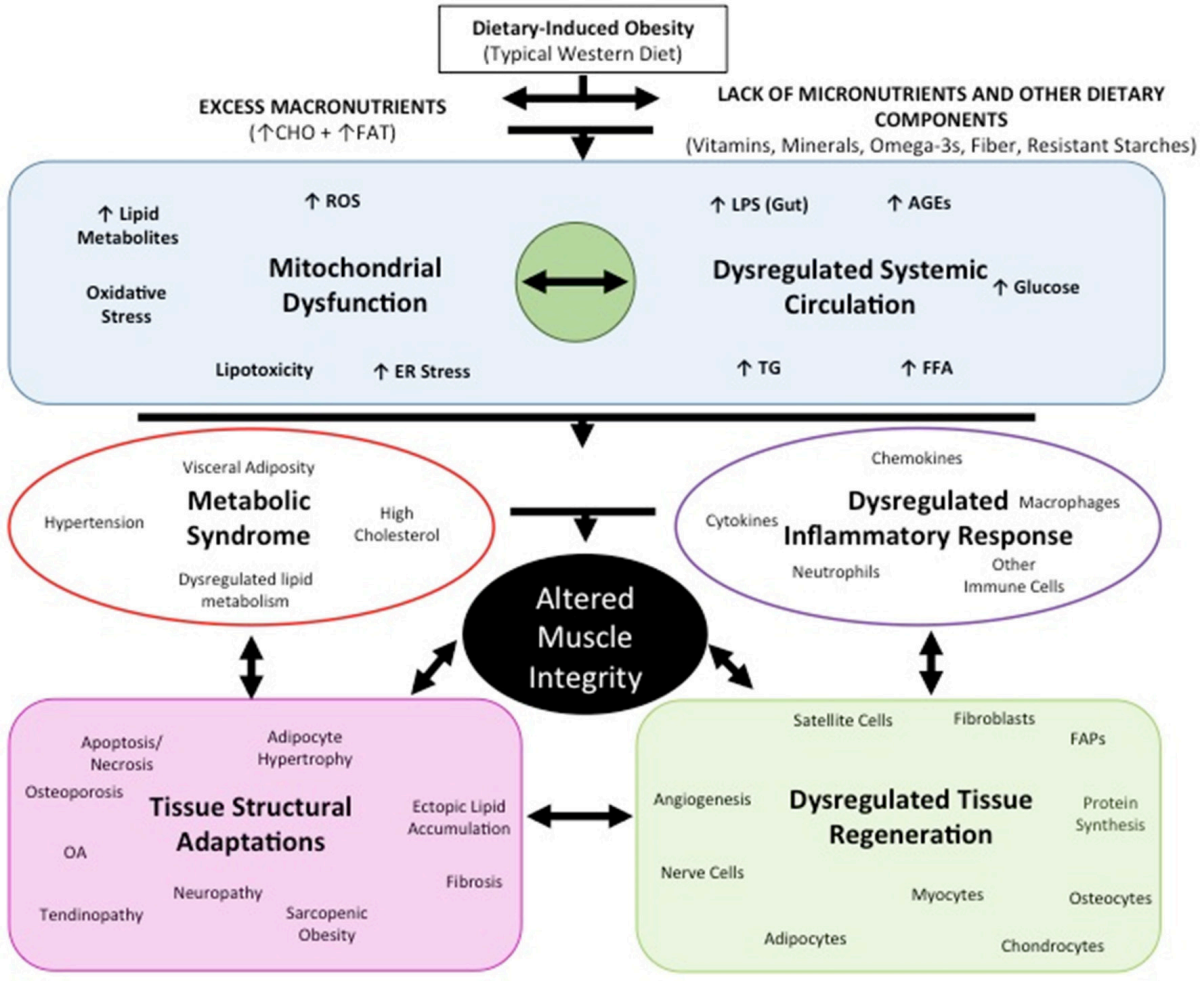
Overview of tissue adaptations and processes with diet-induced obesity.

Once subcutaneous adipose depots become overwhelmed, lipid spill-over leads to ectopic lipid accumulation in visceral adipose tissue depots and other insulin sensitive tissues, such as muscle (Miranda et al., 2005). In muscle, the nutrient overload placed on myocytes, similar to adipocytes, can lead to metabolically dysfunctional cells, further contributing to the already dysregulated inflammatory environment. The complexity and interconnectedness between these processes and mitochondrial dysfunction are illustrated in Figure 7.

## Dietary considerations, nutrient content, and obesity phenotypes

While discussing inflammation from DIO, it is important to consider the composition of the experimental diets, as the dietary profile and energy density of a given diet likely will impact the resultant inflammatory profile in each preclinical model (reviewed in Hariri and Thibault, 2010). For example, diets rich in fat elicit different types of glucose intolerance compared to diets rich in sugar, whereby dietary fat largely compromises muscular glucose tolerance, and sugar challenges liver lipogenic and gluconeogenic enzyme activities (Sumiyoshi et al., 2006). As such, different dietary composition may yield different findings or differences in the severity of disease outcomes.

Fat and sugar dietary constituents can contribute to different animal phenotypes, whereby animals on a high sugar diet do not necessarily gain weight, but demonstrate a conversion of lean body mass to fat body mass (Sumiyoshi et al., 2006). Diets high in saturated fat, however, generally result in increased body fat and body mass, although the type of fat employed (i.e., omega-3 fats) can also have anti-inflammatory effects, and can modulate body fat and body mass (Schemmel et al., 1969; Sumiyoshi et al., 2006; Hariri and Thibault, 2010; Wu et al., 2014, 2017a). To add to the complexity of this matter, many animal species, like humans, demonstrate obesity prone and obesity resistant phenotypes (Schemmel et al., 1969). Arguably, skeletal muscle oxidative metabolism (Shahrokhi et al., 1993) and genetic differences in fat oxidation (Ji and Friedman, 2007; Marrades et al., 2007) may affect whether an animal is prone or resistant to obesity. This feature allows for the experimental evaluation of animals that are exposed to the same amounts of an obesogenic diet, but develop disparate increases in body mass. With MSK disease, this is an important consideration, as tissue damage can be evaluated between animals with similar mass, but increased body fat and low-level inflammation (Hariri and Thibault, 2010). Epidemiological studies reveal MetS can also occur in individuals with typical body weight or that are underweight (e.g., HIV-infected patients), with increases in body fat and metabolic derangement, which underscores the relevance of evaluating obesity resistant animals as a surrogate for this phenomenon to understand MSK diseases in the context of MetS (Drelichowska et al., 2015).

### Nutrient sensing, mitochondrial dysfunction, and metabolic dysfunction

Mitochondria are the primary cellular organelles that generate adenosine triphosphate (ATP). Mitochondrial dysfunction is attributed to excessive nutrient processing, resulting in the uncoupling of oxidative phosphorylation, effectively increasing reactive oxygen species (ROS) generation and decreasing ATP production (Weiss et al., 2013). The free radical theory states that an imbalance between the generation of ROS and antioxidants produced by peroxisomes can result in ROS stealing electrons from other cellular sources, resulting in cellular damage (Ashok and Ali, 1999). Under nutrient overload, as seen in obesity, peroxisomal numbers and/or activity can be impaired as a result of micronutrient deficiencies and/or excessive inflammatory cytokine (e.g., TNF-α) accumulation resulting from elevated oxidative stress and the accumulation of metabolic by-products.

When exposed to nutrient overload, nutrient sensors [i.e., AMP-activated protein kinase (AMPK), sirtuins (SIRT), and mechanistic target of rapamycin, (mTOR)] become impaired, impair mitochondrial function, and can contribute to systemic inflammation, adiposity, and lipotoxicity. AMPK activity also plays a role in bone homeostasis, as an essential mediator of fat and glucose metabolism on bone remodeling (Jeyabalan et al., 2012; reviewed in Finkel, 2015). In skeletal muscle, leptin has been shown to activate AMPK (Salminen and Kaarniranta, 2012), inhibiting FA synthesis, increasing FA oxidation and glucose uptake (Minokoshi et al., 2002). Moreover, limiting nutrient supply and the associated increase in SIRT-1 activity are hypothesized to enhance muscle cell proliferation, while nutrient overload, or age-related loss of SIRT-1, is expected to provide an unfavorable environment for cell proliferation (Akhmedov and Berdeaux, 2013). Likely, reduced cell proliferation occurs, in part, through age-related loss of SIRT, potentially as a consequence of reduced AMPK activity, which may result in loss or dysfunction of mitochondrial activity (Wang et al., 2015; Berenbaum et al., 2017).

In chondrocytes from human OA patients, mitochondrial activity is deficient, leading to a catabolic cascade of responses. Activating AMPK/SIRT-1, mitochondrial master regulator PGC-1a, and FOXO3A mediates chondroprotection, suggesting these pathways may also be critical to maintaining mitochondrial function in chondrocytes, which is critical to preserving cartilage tissue structure (Zhao et al., 2014; Wang et al., 2015; Berenbaum et al., 2017). Mitigating age-related changes in oxidative stress in cartilage (i.e., through SIRT-3) protects against OA-progression and osteoclastogenesis in bone, suggesting that improving the resistance of cartilage to oxidative stress may be one therapeutic target for OA, or for maintaining bone homeostasis (Fu et al., 2016; Huh et al., 2016).

### Reactive oxygen species (ROS) and oxidative stress alterations with obesity

As a by-product of mitochondrial metabolism and homeostasis, ROS are produced and are highly regulated. With cell damage, mitochondrial dysfunction, or oxidative stress, ROS levels accumulate and are associated with the onset of metabolic dysfunction (Serra et al., 2013). ROS accumulation can lead to: lipid peroxidation and disruption of the cellular membrane; ER stress, resulting in protein mis-folding and unfolding, and a decrease in protein synthesis, ultimately rendering cells incapable of clearing misfolded proteins; and activation of Caspase-3 and cell apoptosis (Weiss et al., 2013). Furthermore, oxidative stress in tissues is reported to be an important pathogenic mechanism of MetS onset (Furukawa et al., 2004).

Increased fat mass has been linked to increased systemic markers of oxidative stress in humans and mice (Figure 7) (Furukawa et al., 2004). For example, increased peroxide and reduced endothelial nitric oxide synthase have been associated with cancellous bone loss in an obesity model (Ohnishi et al., 2009). In bone, reactive oxygen species and oxidative stress are critical to osteoclast differentiation. As such, ROS may contribute to osteoporosis and bone catabolism, through activation of RANKL—or receptor activator of NF-κB ligand- influencing osteoclast activity, as well as other pathways downstream such as NF-κB (Callaway and Jiang, 2015). With increased systemic inflammation associated with obesity, bone marrow macrophages and their progenitors can be increased (Singer et al., 2014), with related stimulation of osteoclastogenesis and reduced osteoblast development (Kyung et al., 2009; Halade et al., 2011). Yue and co-workers also reported that leptin produced from obese adipose tissue can bind to leptin receptors on mesenchymal stem cells promoting differentiation to adipocytes and inhibiting osteoblast formation (Yue et al., 2016). The central effects of leptin can also promote cancellous bone loss via the sympathetic nervous system (Ducy et al., 2000; Takeda et al., 2002).

In skeletal muscle, although low-levels of ROS are necessary for force production, high levels of ROS can result in great susceptibility to fatigue and contractile dysfunction (Powers et al., 2011) and are associated with reduced muscle repair capacity (Kozakowska et al., 2015). There may be a link between dysfunctional repair and overuse tendinopathies through ROS production, but this idea remains to be tested experimentally (Bestwick and Maffulli, 2004; Longo et al., 2008). Although the signaling pathway between ROS and osteoarthritis is not clear, there are examples of antioxidant therapy (e.g., dietary polyphenols and hyaluronic acid) that are useful in human studies (reviewed in Lepetsos and Papavassiliou, 2016).

### Lipotoxicity and insulin resistance with obesity

Accumulation of lipids and lipid by-products can result in dysfunction in myocyte metabolic pathways. Lipid concentration is considered a strong indicator of the sensitivity of myocytes insulin-mediated pathways, as well as adipocyte and hepatocyte insulin sensitivity, significantly affecting substrate metabolism and circulating metabolite concentrations (Weiss et al., 2013). The adverse metabolic effects associated with ectopic lipid storage are supported by lipodystrophy studies, showing that muscle lipid accumulation can result in severe insulin resistance and diabetes (Weiss et al., 2013). In addition to the liver, primarily utilized for short term energy storage when circulating nutrient levels are elevated, skeletal muscle is a primary site of glucose uptake and utilization (Yu et al., 2012). Skeletal muscle insulin resistance largely contributes to whole body glucose levels and the presence of a pro-inflammatory environment, as the body is comprised of 40–50% muscle by weight (DeFronzo and Tripathy, 2009; Weiss et al., 2013). In response to low-level inflammation from DIO, extracellular matrix remodeling may contribute to the onset of insulin resistance, thereby inducing collagen synthesis and muscle fibrosis, and contributing to decreased insulin signaling (Williams et al., 2015).

Insulin resistance can also impact tissues of the joint organ system, because insulin resistance has been linked to chondrocyte dysfunction, and insulin appears to have a protective role for synoviocytes, whereby insulin blunts TNF-induced matrix metalloproteinase release (Hamada et al., 2015). However, patients with diabetes exhibit increased synovial levels of TNF-α and macrophages, suggesting that insulin resistance may impair the protective effect of insulin in the joint (Hamada et al., 2016), and greater insulin resistance is related to lower femoral neck strength (Srikanthan et al., 2014).

### Accumulation of metabolic by-products and toxic lipid metabolites in obesity

Elevated levels of oxidative stress can result in incomplete substrate oxidation. These impairments can lead to the excessive accumulation of toxic lipid metabolites (e.g., diacylglycerol, fatty acetyl CoA, and ceramides) and ROS, all natural by-products of cellular metabolism (Broussard and Devkota, 2016). Fatty acid trafficking in muscle may be one of the key factors involved in the onset of insulin resistance, by changing the availability of substrates involved in formation and clearance of harmful lipid intermediates (diacylglycerides and ceramide) (Mittendorfer, 2011). The balance between synthesis and breakdown of these intermediates may influence the balance between the formation and breakdown of FA and TGs, resulting in the storage and development of lipid depots in non-adipose tissues (Mittendorfer, 2011) from alterations in clearance mechanisms.

### Hyperglycemia and advanced glycation end products with obesity

Advanced glycation end products (AGEs) have a pronounced effect on many proteins, particularly collagen. Generally, AGEs accumulate in tissues and form cross-links with targeted proteins, alter cell structure, and interact with receptors that induce oxidative stress and inflammation, resulting in tissue damage (Sanguineti et al., 2014), Figure 5. Specifically, AGEs can alter the physiological failure behavior of some tissues, including tendons (Fessel et al., 2014), by increasing lateral spacing, accumulating cross-links between collagen fibrils, and reducing mechanical properties of tissues by reducing sliding behavior (Gautieri et al., 2017). Moreover, AGEs affect growth and modulate the physiological processes of OA (Franke et al., 2009), and damage-associated molecular patterns (DAMPS) can bind to RAGE and contribute to the pathogenesis of OA (Rosenberg et al., 2017). AGEs can induce muscle atrophy in a RAGE-mediated AMPK down-regulated manner (Chiu et al., 2016) and may affect skeletal muscle growth and contractile function in mice fed a diet high in AGEs (Egawa et al., 2017). In bone, AGEs are associated with decreased bone mineral density and impaired bone quality, eliciting oxidative stress and inflammatory responses in bone cells, while altering material properties of bone collagen fibers via cross-linking (Yamamoto and Sugimoto, 2016).

## Obesity, bacterial lipopolysaccharide (LPS), and gut microbiota

**Figure 8:**
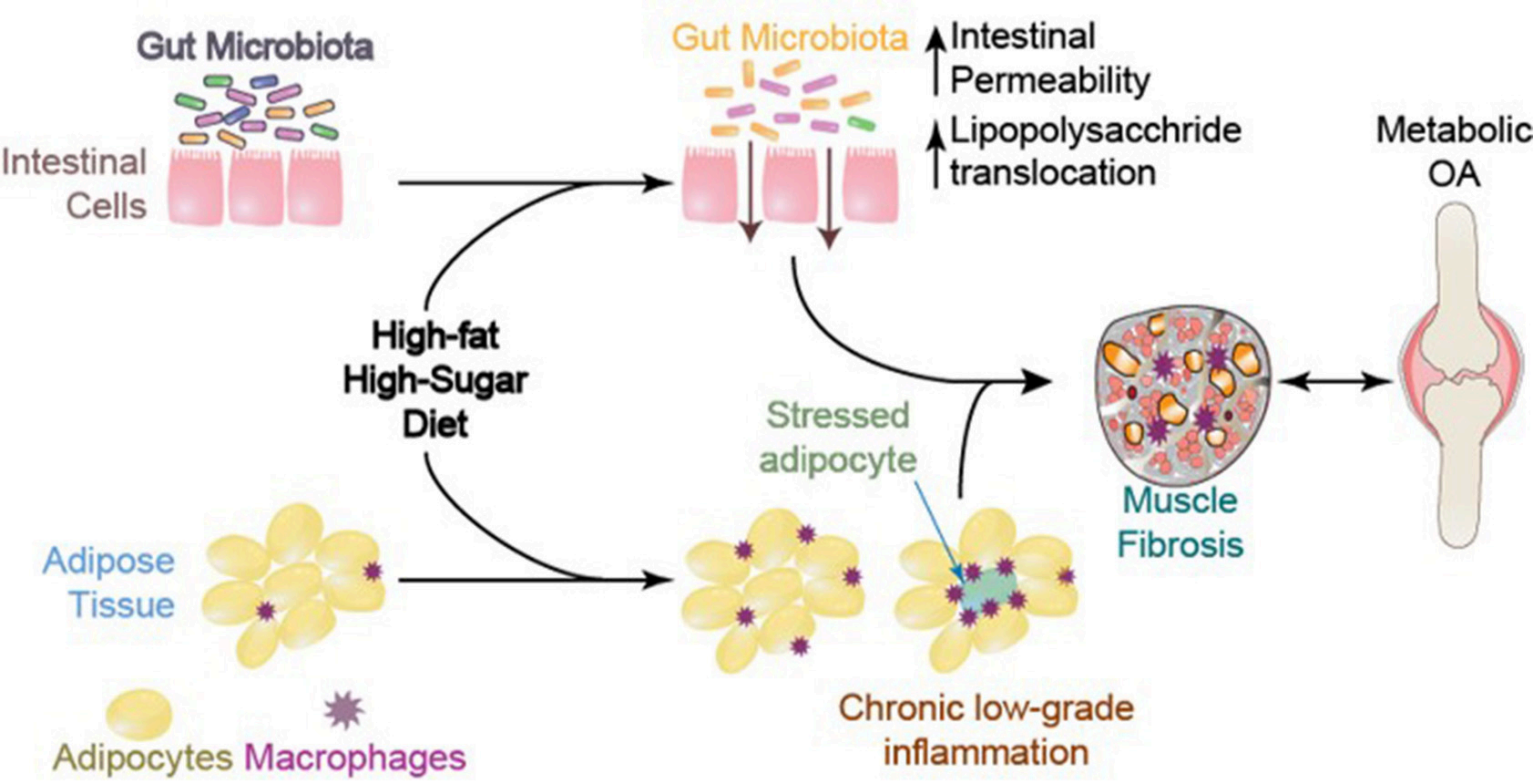
Systemic mediators and compromised muscle integrity are associated with Metabolic OA onset and progression, adapted from Collins et al. (2015a).

It is well-established that the gut microbiota is altered with DIO in a manner that promotes a pro-inflammatory environment (Cani et al., 2009; Cândido et al., 2017). Through changes in tight junction proteins and intestinal barrier integrity, components of gram-negative bacteria may leak into the systemic circulation, resulting in increased systemic LPS concentrations in obese animals including humans (Figure 8) (Cani et al., 2008). Systemic LPS, inflammation, and metabolic disturbance resulting from altered gut microbiota and a leaky gut are believed to be key initiating factors leading to insulin resistance (Cani et al., 2007). In the context of musculoskeletal disease, an impaired gut barrier function has been implicated in rheumatoid arthritis and OA, and may present a viable therapeutic approach for disease management (Scher and Abramson, 2011). However, to date, cause and effect relations between rheumatoid arthritis and the gut microbiota are still being explored (Bravo-Blas et al., 2016).

**Figure 9:**
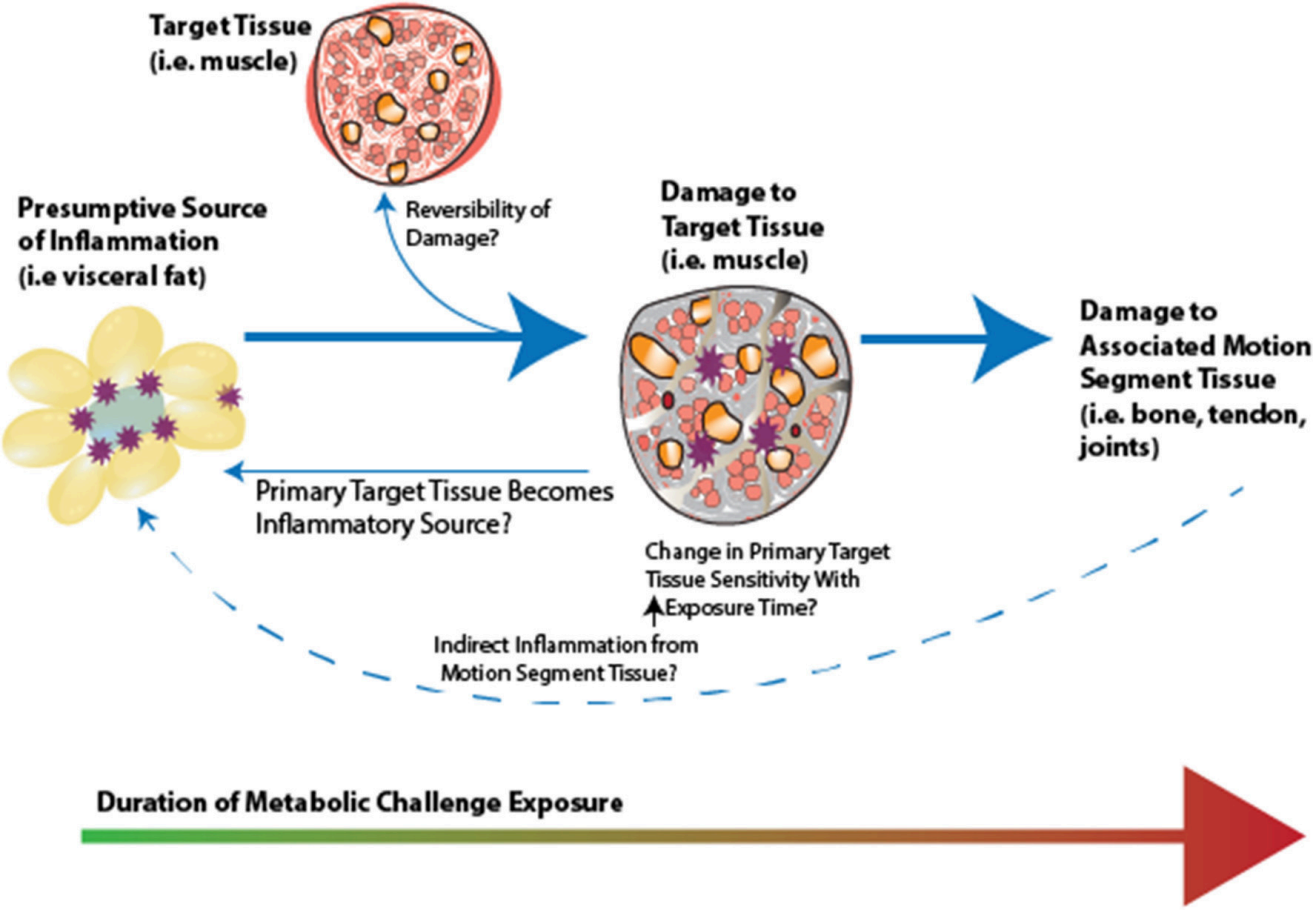
Conceptual framework for inflammatory initiation by a presumptive inflammatory source (i.e., visceral fat, gut microbiota), initiation of adaptation, and subsequent damage in primary target tissue. There is likely a “ripple effect” to the motion segment tissues. Whether associated motion segment tissues becomie inflammatory sources, affecting a presumptive inflammatory source, as well as whether damage is reversible in these tissues, represent interesting open research questions in this area.

We were among the first to identify a significant linkage between constituents of the gut microbiota (*Methanobrevibacter* and *Lactobacillis* spp.) and musculoskeletal changes in metabolic OA/joint damage severity (Collins et al., 2015a) and the onset of compromised muscle integrity (Collins et al., 2016c). These findings link systemic (serum) and local (synovial fluid) to LPS and musculoskeletal changes with diet-induced obesity (Huang et al., 2016) (Figure 9). Using a fecal transplant intervention, lipid profiles can be transferred from donors to germ-free hosts, and altered muscle integrity can be recapitulated, suggesting that the gut microbiota may have a direct effect on muscle development and integrity (Yan et al., 2016). The gut-OA pathophysiological link indicates that dietary and microbiota-based interventions may be important therapeutic opportunities that should be evaluated in the context of MSK health (Collins et al., 2015a; Huang et al., 2016; Berenbaum et al., 2017). There is a need for studies detailing the causal mechanisms of interactions among the gut-microbiota, LPS, and MSK changes. However, given the short-term changes in gut microbiota composition, as well as the decrease in variance in relative abundance across species, studies promoting gut microbiota diversity could be useful in this context. One such candidate is evaluating the impact of prebiotic fiber, which may positively modulate the gut microbiota to promote improved host interactions (Bomhof et al., 2014; Paul et al., 2016; Nicolucci et al., 2017; Parnell et al., 2017).

## Implications for conservative care strategies related to MetS and inflammation

Information regarding pharmacologic management for obesity and metabolic syndrome can be found elsewhere (Apovian et al., 2015), and promising therapeutic targets are discussed throughout this review (and highlighted in Table 3).

**Table 3:** Remaining questions, research agenda, and evidence-supported candidate pathways, targets, and conservative care opportunities.

**Remaining Questions:**
•Is muscle a primary target tissue in the motion segment?
•Do muscular changes directly result in subsequent changes in bone, tendon, cartilage, and joints?
•How does damage in each tissue develop with respect to the other tissues comprising a motion segment?
•What role does MetS/inflammation (and its components) play in manifesting the risks of tissue damage?
•How do the different tissues contribute to homeostasis or damage of the motion segment?
•What is the role in neuroregulation and neuroinflammation in these processes?
**Research Agenda:**
•Evaluate multiple musculoskeletal tissues and diseases in concert
•Evaluate tissues as “sources” or “targets” of low-level systemic inflammation
•Adaptation and impact of sources of inflammation on target tissues are important gaps to address
•Time-course studies are needed to answer these questions
**Evidence-Supported Candidate Pathways:**
•Mitogen-activated protein kinases (MAPK)
•Myeloid differentiation primary response gene 88 (Myd88)
•Nuclear factor kappa-light-chain-enhancer of activated B cells (NFκB)
•NLRP3 inflammasome
**Evidence-Supported Candidate Targets:**
•Adipokines (i.e., Leptin and Progranulin)
•Pro-Inflammatory Cytokines (IL-1B, TNF-a, IP-10)
•SIRT-1
•Reactive Oxygen Species
•Advanced Glycation End Products
•Lipopolysaccharide/TLR-4
•Specialized Pro-Resolving Lipid Mediators
•Follistatin

The inflammatory interface between damage to MSK components and MetS should be a critical consideration, as protecting and preserving MSK structural integrity could be a key approach to conservative management of MetS. Presently, clinical guidelines indicate that weight loss and exercise are good evidence-based conservative approaches for MSK conditions (McAlindon et al., 2014). In the context of osteoarthritis specifically, the combined effect of 18-months of dietary modulation and a combination program involving both aerobic and strength training-based exercise was found to induce weight loss, reduce knee compressive forces, reduce serum IL-6 levels, decrease infrapatellar fat pad size, decrease pain, and increase function in overweight or obese adults with knee OA (Messier et al., 2013; Beavers et al., 2015; Pogacnik Murillo et al., 2017). As such, careful characterization of exercise and dietary interventions in human and other animal models is needed to implement these strategies.

Although there are several candidates for dietary intervention, prebiotic fiber and probiotic supplementation targeting the microbiome (Nicolucci et al., 2017; Parnell et al., 2017), and decreased intake of dietary sugar (Te Morenga et al., 2013) are three safe, readily available, and translatable dietary interventions to protect against MSK damage due to metabolic disturbance that warrant further investigation. As the onset of MSK damage with metabolic disturbance does not have a readily identified starting point, studies are needed to evaluate the efficacy and utility of these dietary modulations in the patient population of interest (e.g., in prevention vs. reversibility and acute vs. chronic), as well as unintended consequences of these modifications. It would be critical to monitor muscle, tendon, bone, and cartilage tissues simultaneously while evaluating these dietary interventions, to facilitate an understanding of the benefits and interconnectedness of these MSK tissue changes and diseases.

## Opportunities and future research directions

There are several gaps in the current understanding of the effects of low-level systemic inflammation vs. metabolic disturbance on musculoskeletal health. Many of these gaps are summarized in Table 3. Whether muscle is a primary target tissue in the motion segment, and whether muscular changes directly result in subsequent changes in bone, tendon, and joints, remains to be assessed and clarified. The studies presented here underscore the need for evaluating multiple musculoskeletal tissues and diseases in concert with MetS (as well as defining which elements of the MetS are responsible for which consequences of obesity). This approach will facilitate an understanding of how damage in each tissue develops with respect to the other tissues comprising a motion segment, how the different tissues may contribute to homeostasis or damage of the motion segment, and what role that MetS/inflammation plays in manifesting the risks of tissue damage. At present, it is unclear if: MetS-related inflammation increases in severity with chronicity; systemic inflammation is constant with continued metabolic disturbance; or MetS-related inflammation is dynamic and fluctuating. It may be useful to evaluate tissues as “sources” or “targets” of low-level systemic inflammation (Figure 9). This conceptual framework is demonstrated in the data from our previous studies, which suggest a systemic-to-local time-course shift from serum to synovial fluid, and serum to muscle changes (Collins et al., 2016a,b). Briefly, metabolic dysregulation initiates an inflammatory cascade, whereby an early perturbation in a presumptive inflammatory source (i.e., visceral fat and gut microbiota) results in target tissue (i.e., muscle) adaptation and subsequent damage. Although the source of the inflammation is unclear, as are the specific pathways involved, target tissue inflammation and damage may affect subsequent associated motion segment tissues (i.e., bone, tendon, and joints), and these relations are affected by many factors (i.e., the dynamic make-up/profile of the inflammatory components, time of exposure, and severity of the metabolic disturbance).

Furthermore, adaptation and impact of sources of inflammation on target tissues are important knowledge gaps that need to be addressed. It would be of value to determine if metabolic dysfunction and regulation are integrated and interdependent, or if source tissues and target tissues of the motion segment are regulated and affected differently and independently, at least in a first approximation. Identifying the potential for reversibility of tissue damage with diet-induced obesity is another critical area of research that should be addressed. The mechanism(s) by which target tissue or motion segment tissue changes may contribute to MetS and inflammation are incompletely understood at this time. Time-course studies are needed to answer these questions.

Discussion of the impact of metabolic disturbance on neuroregulatory systems, and a role for neuroinflammation-mediated influence on loss of musculoskeletal integrity was limited in this review, but this may be a potentially important area for future research. Nearly all of the tissues of a motion segment discussed are innervated except for adult cartilage, and thus, neuro-regulation and how it is affected primarily and secondarily by MetS associated inflammation, may be critical in understanding the underlying mechanistic considerations. In particular, how these changes in neuroregulation and neuroinflammation contribute to a loss of muscle integrity is an interesting area for future research.

## Summary

Studies presented in this review implicate several pathways that may be critical for the onset and progression of systemic inflammation due to metabolic disturbance and musculoskeletal damage. Some of the pathways that could be involved are mitogen-activated protein kinases (MAPK, p38 MAPK, JNK), myeloid differentiation primary response gene 88 (MyD88), NFκB, and the NLRP3 inflammasome. Likely, some or all of these pathways may be activated in parallel creating a potentially positive-feedback based redundant system. Investigations targeting specific pathways in the context of metabolic disturbance and MSK disease will provide much needed mechanistic insights to better understand the consequences of diet-induced obesity.

A motion segment, such as a leg is comprised of a number of interdependent components (i.e., muscles, tendons, bones, and articular joints) that rely on the integrated biological and mechanical integrity of all tissues for optimal function. Thus, the direct and indirect impact of dysregulation via MetS and associated inflammation on any one element may have a “ripple” effect that may be compounded over time. There are many important and relevant gaps in this research area, and addressing them will provide valuable insights into the relations among the motion segment, common inflammatory pathways, and resulting MSK disease.

## Author contributions

KC was responsible for conception and design, collection, and assembly of data analysis and interpretation of the data, drafting of the article, critical revision of the article for important intellectual content and final approval of the article. WH, GM, RR, JR, IS, and RZ were responsible for conception and design, collection and assembly of data analysis, interpretation of the data, critical revision of the article for important intellectual content and final approval of the article. DH was responsible for conception and design, analysis and interpretation of the data, drafting of the article, critical revision of the article for important intellectual content and final approval of the article.

### Conflict of interest statement

The authors declare that the research was conducted in the absence of any commercial or financial relationships that could be construed as a potential conflict of interest.
